# New Year’s Res-Illusions: Food Shopping in the New Year Competes with Healthy Intentions

**DOI:** 10.1371/journal.pone.0110561

**Published:** 2014-12-16

**Authors:** Lizzy Pope, Andrew S. Hanks, David R. Just, Brian Wansink

**Affiliations:** Food and Brand Lab, Charles H. Dyson School of Applied Economics and Management, Cornell University, Ithaca, New York, United States of America; The University of Kansas Medical Center, United States of America

## Abstract

**Objective:**

How do the holidays – and the possible New Year’s resolutions that follow – influence a household’s purchase patterns of healthier foods versus less healthy foods? This has important implications for both holiday food shopping and post-holiday shopping.

**Methods:**

207 households were recruited to participate in a randomized-controlled trial conducted at two regional-grocery chain locations in upstate New York. Item-level transaction records were tracked over a seven-month period (July 2010 to March 2011). The cooperating grocer’s proprietary nutrient-rating system was used to designate “healthy,” and “less healthy” items. Calorie data were extracted from online nutritional databases. Expenditures and calories purchased for the holiday period (Thanksgiving-New Year’s), and the post-holiday period (New Year’s-March), were compared to baseline (July-Thanksgiving) amounts.

**Results:**

During the holiday season, household food expenditures increased 15% compared to baseline ($105.74 to $121.83; *p*<0.001), with 75% of additional expenditures accounted for by less-healthy items. Consistent with what one would expect from New Year’s resolutions, sales of healthy foods increased 29.4% ($13.24/week) after the holiday season compared to baseline, and 18.9% ($9.26/week) compared to the holiday period. Unfortunately, sales of less-healthy foods remained at holiday levels ($72.85/week holiday period vs. $72.52/week post-holiday). Calories purchased each week increased 9.3% (450 calories per serving/week) after the New Year compared to the holiday period, and increased 20.2% (890 calories per serving/week) compared to baseline.

**Conclusions:**

Despite resolutions to eat more healthfully after New Year’s, consumers may adjust to a new “status quo” of increased less-healthy food purchasing during the holidays, and dubiously fulfill their New Year’s resolutions by spending more on healthy foods. Encouraging consumers to substitute healthy items for less-healthy items may be one way for practitioners and public health officials to help consumers fulfill New Year’s resolutions, and reverse holiday weight gain.

## Introduction

How do the holidays – and the possible New Year’s resolutions that follow – influence a household’s purchase patterns of healthier vs. less-healthy foods? Holiday weight gain has been largely documented, but less understood is the potentially compensating cycle of food purchasing that might start much earlier in the season and run much later. Food purchases dictate the total calories available in the home and over-purchasing can have a long-term impact on weight gain that would not be reflected in short-term studies of holiday weight gain. During the holiday season, weight gains of 0.37 kg to 0.93 kg do occur, and may not be reversed after the holidays are over [1]–[5]. Although, the weight gained during the holiday period may seem inconsequential, it actually can account for a large proportion of the small yearly weight gains of 0.5–1.0 kg documented in adults, as New Year’s resolutions to lose these pounds are not generally successful [2], [6], [7]. The small yearly weight gains seen in American adults have been hypothesized to be responsible for the gradual increase in obesity rates since the 1970’s [8]–[10]. With these holiday weight gains in mind, it is interesting to consider how food purchases may change during the holidays, and how they may shift after the holidays.

It is widely assumed that people increase junk-food purchasing and consumption during the holidays and there are several behavioral factors that may explain why individuals are caught overindulging. Longer eating durations, eating with others, easy access to foods, and increased portion sizes are all prevalent during the holidays, and have all been associated with increased intake [11], [12]. People’s eating environments are often altered during the holiday period as they attend more parties, eat with friends more frequently, are exposed to leftovers, and have to manage distractions concurrent with making eating decisions [11]. Food environments also often shift during the holiday period with food becoming more salient, portions larger, and stockpiles abundant [11]. Additionally, stress levels frequently increase during the holidays, leading people to have an even more difficult time making healthy-eating decisions and resisting the constant temptations of the holidays [13]. All of these influences on our eating and food environments are potential reasons why food purchasing and food consumption may increase during the holiday season.

After the holidays, New Year’s resolutions to reach the “ideal weight” are often quickly abandoned as individuals return to previous habits [6], [14]. But do individuals settle back into pre-holiday shopping patterns, or did the holiday frenzy establish a new status quo of food purchasing that may undermine New Year’s resolutions to eat healthier? While several studies have recorded general household food purchasing patterns, seasonal shifts in purchasing have not been documented [15]–[17]. Although food purchasing does not directly measure food consumption, previous research has shown a strong correlation between receipts recording food purchases and dietary recalls reflecting consumption in terms of total fat, total calories, and percent calories from fat [18], [19]. The objective of the current study was to examine the food purchasing behavior of 207 households over the holiday period. Purchase patterns before, during and after the holiday season were compared. Purchasing data was also used to compute the number of calories purchased and dollars expended on healthy and relatively less-healthy foods before, during, and after the holidays.

## Methods

### Ethics Statement

Participants provided written informed consent before purchase-monitoring began, and the study was approved by the Cornell University Institutional Review Board.

### Participants and Data

This study was conducted as part of a larger seven-month study on purchasing patterns of healthy and relatively less healthy foods. In order to designate healthy and relatively less healthy foods, the research team identified a grocer in the Northeastern United States that had developed a proprietary health rating system for their products. This system, know as Guiding Stars, utilizes a scoring algorithm to categorize foods and beverages into one of four groups–zero, one, two, and three stars–with three stars being the most nutritious [20]. The algorithm is based on the nutrients per calorie that foods provide, and rates foods from zero (less-healthy) to three (healthy) “stars.” For example, soda receives zero stars, whereas 1% milk receives three stars. This particular rating system captures most foods and beverages available at the grocery store but does not account for food items with zero calories, such as water and diet soda, as well as promotional or seasonal items that are sold for a limited time. Thus, these particular foods and beverages do not receive a rating.

Participants for the study were recruited during the months of June and July 2010 in three stores located in a Western New York city with 62,235 residents. Individuals were notified of the study through face-to-face public intercept, emails, word of mouth, and flyers posted in the grocery stores. Shoppers who did more than 75% of the household shopping were targeted so the study would capture a more representative picture of purchasing for an entire household. In addition, shoppers completed a demographic survey assessing income, age, education level, employment status, family size, and marital status.

As an incentive for participation, individuals received a 10% discount on purchases of rated items (including zero starred items), which was loaded onto a Bank of America debit card at the end of each week. Each participant received a specific ID card, which was scanned during every transaction. The grocer separated these transactions and sent the data to the research team. With this data, the research team determined the participant’s reimbursement and loaded the reimbursement onto the debit card once a week. This 10% discount lasted during the baseline data collection period from July 17 through September 6.

Over the course of 37 weeks, from July 17, 2010 to March 12, 2011, daily-itemized transaction level data were collected for 207 participating households. Each household had a specific identification code, which the grocer used to separate transaction records. These records were then sent to the research team via secure electronic transmission. Data in the transaction records included the health rating of each item purchased, price, product descriptions, total quantity of each item purchased, and total expenditures. With this data, groupings of products by health-rating, “healthy” or “less-healthy,” could be constructed.

Additionally, the grocer utilized a category code to classify its approximately 20,000 products. This category code contained nearly 1000 groups into which specific items were classified. In order to obtain nutrition information, the highest frequency item purchased within these product groups was used to extract calorie, fat, and sugar information from online nutrition databases and manufacturer websites. These data were then merged to the transaction data for analysis.

### Analysis

In each household, data were aggregated each week, Sunday through Saturday, such that one outcome measure of interest is weekly household food expenditures. Since we utilized the participating grocer’s proprietary rating system we identified healthy foods as receiving at least one star, and relatively less healthy foods as receiving no stars. We then separated expenditure data into purchases of healthy and relatively less healthy foods.

The second outcome measure utilized in analysis was weekly per-serving calorie aggregates. This measure was generated by generating a calorie per-serving value for each item purchased and summing these per-serving calories by week for each household. We used a per-serving measure since this is the information available on food labels. In addition, this measure standardizes caloric amounts for all food items making comparisons much more transparent.

In the analysis, we used ordinary least squares regression to estimate the difference of holiday and post-holiday purchasing when compared to a baseline level. We refer to the period lasting from July 17 to November 13, 2010 as the baseline period, the period lasting from November 14, 2010 through January 1, 2011 as the holiday season, and the period lasting from January 2 to March 12, 2011 as the post-holiday season. In the regression analyses we controlled for treatment condition received in a larger study conducted concurrently, age, BMI, income, education, and number of children in the household.

## Results

**Figure 1:**
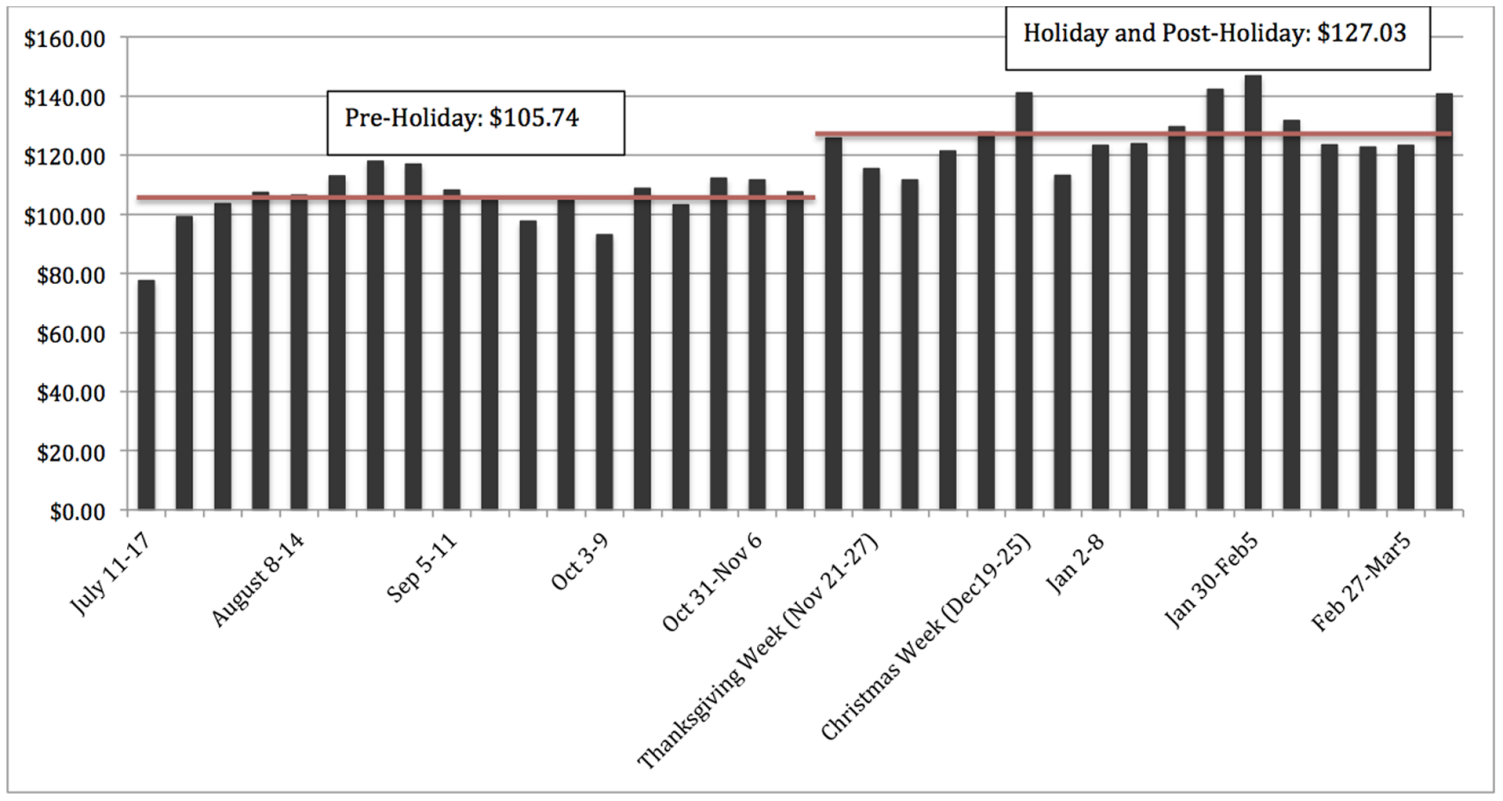
Average Weekly Expenditures Increase During Both the Holiday and Post-Holiday Seasons. Weekly average expenditures were plotted out for the duration of the study. Thanksgiving fell on week 20 of the study and Christmas fell on week 24.

**Figure 2:**
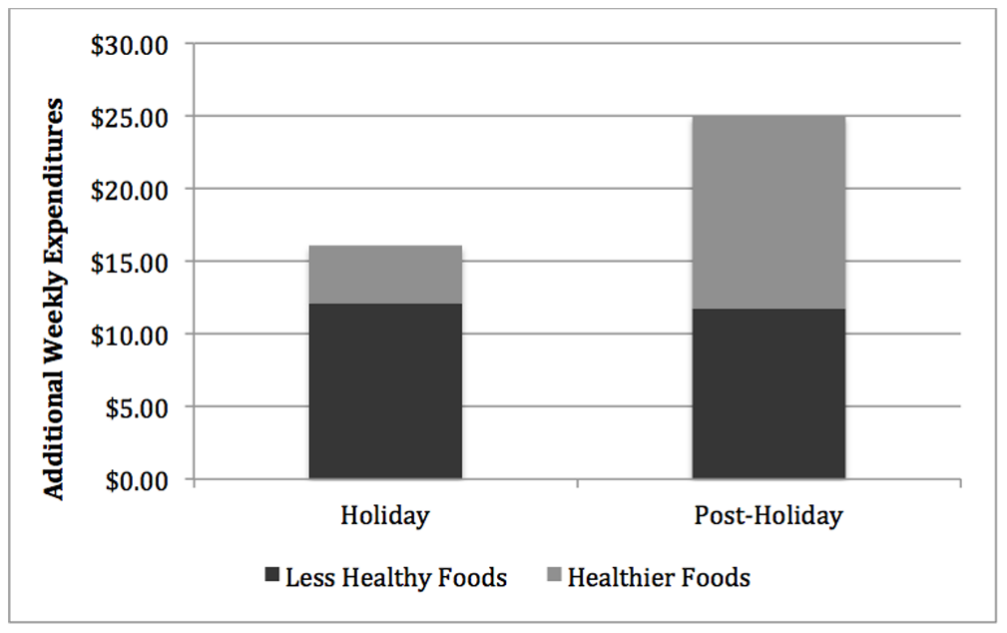
Households Increase the Purchase of Healthier Foods After the Holidays. Additional expenditures were calculated by subtracting expenditures in the holiday and post-holiday seasons from baseline expenditures. Additional expenditures for the less healthy and healthier items were stacked to show total additional expenditures in the respective period.

Average household weekly (Sunday through Saturday) expenditures shown in Figure 1 suggest a level shift in expenditures that occurred one week prior to Thanksgiving (week 19) through the end of the study. Interestingly, this shift appears to have persisted throughout the duration of the study, with peaks at week 24 (includes Christmas) and week 30 (week prior to Super Bowl Sunday). To quantify the level shift in expenditures and calories evident in Figure 1, we report regression results that separated the data into the baseline, holiday, and post-holiday season. Regression results (Table 1; Figure 2) indicate that, when compared to baseline levels, households in the sample spent an additional $16.09 per week (*p*<0.001) on food and beverage items with $12.11 (*p*<0.001) spent on less healthful foods and $3.98 (*p*<0.05) spent on healthier items during the holiday season. About 75% of additional expenditures were dedicated to less healthful food items.

**Table 1:** Predicted Average Expenditures and Calories During the Baseline, Holiday, and Post-Holiday Periods.

	Baseline	Holiday Season	Post-Holiday Season	F-statistic
	(std. err.)	(std. err.)	(std. err.)	(p-value)
Expenditures				
Expenditures	$105.74	$121.83	$130.76	23.300
	(2.072)	(3.263)	(2.808)	(0.000)
Non-Starred Exp	$60.74	$72.85	$72.52	27.030
	(1.109)	(2.094)	(1.636)	(0.000)
Starred Exp	$45.00	$48.98	$58.24	29.850
	(0.839)	(1.360)	(1.324)	(0.000)
Calories	4396.465	4836.611	5286.282	22.740
	(78.250)	(126.290)	(111.502)	(0.000)
Non-starred Cals	2874.743	3274.956	3435.008	23.250
	(56.118)	(93.145)	(79.407)	(0.000)
Starred Cals	1521.722	1561.654	1851.274	17.710
	(29.150)	(43.450)	(41.667)	(0.000)

Values in this table are based on predicted means from an ordinary least squares regression where expenditures were the outcome variable. A variable indicating purchase periods, baseline, holiday season, and post-holiday season, was the independent variable of interest. We also controlled for age, bmi, income, education, and number of children in the household. The F-statistic is from the regression and indicates overall model explanatory power. Standard errors and p-values are in parentheses.

In the post-holiday period, households continued the upward trend in expenditures and spent an additional $25.01 per week (*p*<0.001), a 23.7% jump in weekly expenditures compared to the baseline period. Households spent about the same on the less-healthy items in the post-holiday season as they did during the holiday season ($11.77; *p*<0.001). However, when compared to baseline levels, households increased expenditures on the more nutritious foods by an estimated $13.24 (*p*<0.001) after the holidays. This more than triples the $3.98 increase in expenditures seen in the holiday season (*p*<0.001).

Interestingly, the mix of foods purchased during the holiday season shifted in favor of the less healthy foods as the share of expenditures dedicated to these foods increased from 57.1% during the baseline period to 59.3% during the holiday period (*p* = 0.006). Yet in the New Year these gluttonous foods accounted for 55.7% of expenditures, a decrease of 1.4 percentage points from baseline (*p* = 0.057). Since expenditures on the more calorically-dense foods remained flat, this drop in expenditure shares reflects the increase in expenditures on the healthier items in the post-holiday period. While additional expenditures on the more nutritious items in the post-holiday period nearly tripled additional expenditures in the holiday period, households were actually spending $0.53 more per item on these foods in the post-holiday period than the holiday period. Thus in the New Year, households tended to seek out the more expensive versions of the healthier items instead of seeking the best value.

**Figure 3:**
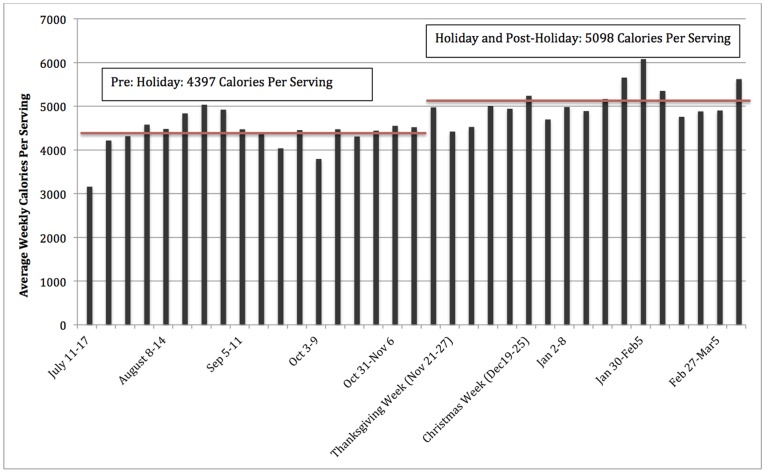
Households Purchase More Calories (per Serving per Shopping Trip) During the Holiday and Post-Holiday Seasons. Weekly average calories purchased were plotted out for the duration of the study. Thanksgiving fell on week 20 of the study and Christmas fell on week 24.

**Figure 4:**
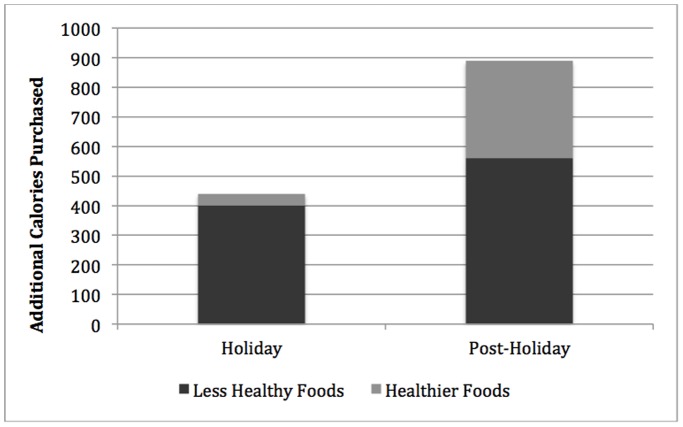
Households Purchased Additional Calories of Healthier Foods During the Post-Holiday Period. Additional calories were calculated by subtracting calories in the holiday and post-holiday seasons from baseline expenditures. Additional calories for the less healthy and healthier items were stacked to show total additional calories in the respective period.

Given these purchasing patterns, were households stocking up with more calories during the holiday and post-holiday periods (Table 1; Figures 3,4)? Total weekly per-serving calories increased by 440 (*p*<0.0.003) during the holiday period relative to the baseline period, and nearly 91% of this increase was due to additional purchases of the more calorically-dense foods. For the healthier foods, the increase in total weekly per-serving calories purchased was not significant at conventional levels suggesting a strong focus on the more fattening foods.

Even more intriguing is that contrary to well-intentioned New Year’s resolutions, additional weekly per-serving calories purchased increased to 890 in the post-holiday period (*p*<0.001) relative to baseline, more than doubling the 440 calorie increase evident in the holiday season. Of this 890 calorie increase, 560 calories were attributed to the calorically-dense foods while healthier items made up the additional 330 calories, suggesting a greater focus on healthier items post-New Year’s Day. Yet, since expenditures on the less-healthy foods remained constant, households may have been purchasing less-healthy foods with higher calorie content.

When we compared additional weekly per-serving calories purchased of healthy foods from the holiday and post-holiday seasons, we found that total weekly per-serving calories purchased jumped from 40 (*p*<0.453) in the holiday period to 330 (*p*<0.001) in the post-holiday period, a 725% (*p*<0.001) jump. This increase in healthy-item purchasing from the holiday period to the post-holiday period accounts for over half of the additional 450 (440 to 890) calories purchased in the post-holiday period compared to the holiday period. In other words, additional weekly per-serving calories for each healthy item purchased increased slightly by 10 calories in the post-holiday period, indicating that households were purchasing more expensive *and* somewhat more calorically dense “healthier” items.

## Discussion

As might be expected, during the holiday season individuals spent more money on food and purchased more weekly per-serving calories than during the summer/fall. Our findings agree with previous research which has also shown increased calorie intake during the holiday season due to consumption of more energy dense foods as well as purchasing for larger groups [21], [22]. During the holiday period, less-healthy foods contributed to 91% of the additional weekly per-serving calories purchased, so the caloric increase was not from nutrient-dense foods. Most interestingly, this increased food and calorie purchasing persisted after the New Year where individuals spent 55% more on food and purchased twice as many additional weekly per-serving calories than during the holiday period.

While expenditures on the healthier starred foods increased by more than nine times, and total weekly per-serving calories purchased from starred foods rose by 725% in the post-holiday period, households maintained the level of non-starred item purchasing they had adopted during the holiday period. Consequently, if households resolved to eat more healthfully in the New Year, they may have fulfilled this desire not by decreasing purchases of less-healthy items or total weekly per-serving calories, but by buying fewer, but more expensive and slightly more calorically-dense healthy items. Because holiday food purchases are generally driven by tradition versus health concerns, it is possible that participants remained in a state of willful ignorance surrounding the nutrition content of their holiday purchases [23].

The finding that after New Year’s purchasing of healthy items increased and relatively less-healthy items remained the same as during the holiday period suggests that even though many people make a New Year’s resolution to eat healthier or lose weight, consumers are making purchasing decisions that only partly support these goals. The sustained increased purchasing seen after New Year’s may be due to the status-quo bias, which suggests that even when people recognize that making a change would be best for them, they still continue to follow their behavioral scripts [24]. The period between Thanksgiving and Christmas may establish a new purchasing “status quo” for people in terms of both money spent and calories bought. The uptick in purchasing in general and purchasing of non-starred items in January compared to September may suggest that people are habituated to new baseline purchasing patterns, and may have trouble reverting to their early-fall less calorific purchasing.

Because the study did not track a full year of food purchasing, it is impossible to see when purchasing might return to pre-holiday levels. One hypothesis is that as winter ends, and people start gearing up for summer and “bikini season,” food purchasing decreases and establishes the pre-holiday baseline observed in our study from July to November. Although little work has been done elucidating the seasonality of weight gain/loss, a study of advertising in popular women’s magazines suggested that dieting themes were dominant during the summer months [25]. These results support our hypothesis that spring/early summer is the most likely time for food purchasing to decrease. It would be interesting to determine whether any potential spring/summer scale back in purchasing is large enough to counteract the increase in calories purchased during the holiday and post-holiday periods. In other words, does this period serve as a good “reset” point, or does purchasing continue to rise slightly year after year, never quite returning to the level from the previous year? If food purchasing and calories purchased do continue to rise each year, this could contribute to small yearly weight gains, which eventually can lead to overweight and obesity. Future studies could track purchasing over a full year to see a complete yearly cycle of food purchases.

In addition to adjusting to a new purchasing status quo, increased purchasing of healthier items, and sustained elevated purchasing of non-starred relatively less healthy items in the post-holiday period may indicate that people were experiencing the impact of a “health halo.” Previous research has illustrated that low-fat claims on foods led to greater consumption of those foods by reducing consumption guilt and increasing perceived serving size [26]. In the present study, increased purchasing of starred foods may indicate that consumers felt they could consume greater quantities of those foods with reduced guilt. Furthermore, Wilcox et al. discovered that when a healthy item was added to someone’s choice-set of foods to consume, people actually increased their consumption of indulgent foods. Apparently, by even considering the healthy food as a choice, people vicariously fulfilled their goals to eat healthfully, and then actually chose a more-indulgent item to consume [27]. It is possible in the current study that merely purchasing the healthier items formed a sort-of “health halo” which dubiously fulfilled participants’ goals to purchase healthy foods, and relieved them of any guilt associated with purchasing non-starred items.

### Limitations and Future Research

Limitations to the study include the fact that the study tracked purchasing for a household, and not actual food consumption. However, previous research has indicated that purchasing records such as receipts provide an accurate record of actual consumption [18], [19]. Furthermore, we did not measure change in weight or other anthropometric variables, so we do not know whether the increased purchasing led to weight gain as had been reported in previous research. Future studies could track both purchasing behavior and weight change over the holiday period. Next, we did not track per-unit price changes, so we don’t know if holiday discounts may account for the increased expenditures seen in the holiday period vs. the post-holiday period. However, the weekly presence of markdowns and specials in the grocery store environment suggests that although prices of some items may have increased after the holidays, others may have decreased and vice versa. Because participants were all given a 10% discount on rated items to participate in the study, it is possible that purchasing was confounded by this. However, since we wanted to use a nutrient rating system to help determine whether purchasing was “healthy” or “unhealthy” it was important to promote purchasing of rated items. We also point out that our data do not include food away from home purchases or account for the fact that during the holidays, children are at home and not at school. Yet, even when children return to school in the New Year, food expenditures are still higher compared to the pre-holiday season, though this is mainly true for the healthier foods. Finally, since calorie data was not pulled based on UPC data, but rather the most frequently purchased item within a food group, the calorie estimates are only approximations. While these estimates and can be improved upon in future research with individual product information pulled using UPC codes, this process can be costly.

Strengths of the study include its long duration, which allowed examination not only of holiday purchasing, but also of pre- and post-holiday purchasing. Although the study was not able to track all of spring/summer purchasing, it still gives a very interesting picture of food purchasing expenditures over ¾ of the calendar year. The study is also the first to elucidate purchasing differences between “healthy,” and “less-healthy” items over the holiday period, providing interesting insight into how the six-week holiday season impacts not just overall purchasing, but specific categories of items. Results suggest that future research could focus on ways to help consumers decrease their purchasing of less nutrient-dense items once the holiday season is over.

### Implications

Small yearly weight gains of one to two pounds may be a large contributor to the high rate of overweight and obesity in America [2]. The fact that weight gain over the holiday period may be responsible for half of this yearly weight gain suggests that examining eating and food-purchasing patterns over the holidays is important [1]–[5]. The current study found that not only did purchasing of less-healthy items increase during the holiday season, but it remained elevated in the weeks immediately following this season. Although consumers also increased purchasing of healthy items post-holidays, these items appear to have provided a “health halo,” or vicarious goal fulfillment especially during the time period, after New Years, when many Americans resolve to eat more healthfully.

Several strategies may be useful in controlling the increased food purchasing seen both during the holidays and after the holidays. Pre-determined shopping lists may deter impulsive purchasing at the grocery store, which tends to result in purchases of less nutritious foods [13]. Furthermore, helping shoppers remember what their purchasing patterns looked like in the early fall before the holiday season began might be a good way to help them return to their “pre-holiday” status quo, and not remain at their “holiday” status quo. Finally, using visual cues that divide shopping carts and baskets in half and encourage the consumer to fill half the cart with high-nutrient items may be one way to increase consumption of healthier foods, while simultaneously restricting the number of “junk food” items purchased to half the cart [28]. Combining these strategies with realistic weight-loss goals may lead individuals to successfully shed unwanted pounds, and even keep them off [14], [29], turning their New Year’s illusions into successful resolutions.
